# Effectiveness of Mental Health Warnings on Tobacco Packaging in People With and Without Common Mental Health Conditions: An Online Randomised Experiment

**DOI:** 10.3389/fpsyt.2022.869158

**Published:** 2022-07-14

**Authors:** Katherine Sawyer, Chloe Burke, Ronnie Long Yee Ng, Tom P. Freeman, Sally Adams, Gemma Taylor

**Affiliations:** ^1^Addiction and Mental Health Group (AIM), Department of Psychology, University of Bath, Bath, United Kingdom; ^2^Institute for Mental Health, University of Birmingham, Birmingham, United Kingdom

**Keywords:** tobacco warning labels, tobacco control, mental health, smoking, survey

## Abstract

**Background:**

Health warning labels on tobacco packaging are a cost-effective means of health risk communication. However, while an extensive range of physical health risks are well-portrayed *via* current tobacco health warnings in the UK, there are none that currently portray the negative impact of smoking on mental health.

**Aims:**

(i) develop novel mental health warning labels for tobacco packaging and (ii) test perceptions of these warnings in smokers and non-smokers, with and without mental health problems.

**Methods:**

Six mental health warning labels were developed with a consultancy focus group. These warning labels were tested in an online randomised experiment, where respondents (*N* = 687) rated six Mental Health Warning Labels (MHWLs) and six Physical Health Warning Labels (PHWLs) on measures of perceived effectiveness, believability, arousal, valence, acceptability, reactance and novelty of information.

**Results:**

MHWLs were perceived as low to moderately effective (mean = 4.02, SD = 2.40), but less effective than PHWLs (mean = 5.78, SD = 2.55, *p* < 0.001, η*_*p*_*^2^ = 0.63). MHWLs were perceived as less believable, arousing, unpleasant, and acceptable than PHWLs. MHWLs evoked more reactance and were rated as more novel. Perceptions of MHWLs did not differ in people with and without mental health problems except for reactance and acceptability, but consistent with the PHWL literature, perceptions of MHWLs differed between non-smokers and smokers.

**Conclusion:**

MHWLs could be an effective means to communicate novel information about the effects of smoking on mental health. MHWLs are perceived as less effective, believable, arousing, unpleasant, and acceptable than PHWLs, but MHWLs evoke more reactance and are rated as more novel.

## Introduction

Smoking is the leading cause of preventable death and illness in the UK (1), with 77,800 deaths per year estimated to be attributable to smoking (2). In high income countries, smoking rates have declined among the general population (3, 4). However, people with common mental health conditions such as anxiety and depression, are twice as likely to smoke than the general population (5, 6). Smokers with mental health conditions encounter substantial barriers to cessation, such as heavier smoking and greater nicotine dependence and withdrawal symptoms (7–10). People with mental health conditions die 10–20 years younger than the general population, and smoking is a primary reason for this (11, 12). Smoking represents a major driver of health inequalities and there have been calls from governments and healthcare agencies for bespoke and targeted interventions for people with mental health conditions (4, 13).

It is well established that smoking damages physical health, and warning labels on tobacco packaging are a cost-effective method of communicating these health risks (14). In 2017, the UK implemented plain tobacco packaging with pictorial warnings (15). There is an extensive range of physical health risks portrayed on tobacco health warning labels in the UK and internationally (16), which are demonstrated to be effective in promoting smoking cessation, and reducing smoking uptake (14, 17, 18). However, it is less well known amongst the general public and healthcare professionals that smoking can negatively affect mental health (19–21). A large body of evidence suggests that tobacco use increases the risk of developing depression, schizophrenia and bipolar disorder (22–25), and that smoking cessation can reduce symptoms of depression, anxiety, and stress, and lead to improved wellbeing and positive feelings (26). Qualitative research suggests that people who smoke and have mental health conditions “buy in” to the idea that tobacco can worsen mental health, they understand that smoking can make their depression and anxiety worse, and that quitting could improve their mental health (27). Hence, mental health warnings on tobacco packaging represent a key strategy to promote smoking cessation and prevent uptake, by, for example, increasing understanding and believability of the link between smoking and mental health, or increasing arousal when viewing tobacco warning labels.

The World Health Organisation (WHO) recommends that warnings on tobacco packaging should expand to include the risks of smoking for mental health (28), however, only one country has adopted this recommendation, and there is only one study testing the effectiveness of one MHWL. Columbia introduced one mental health warning in 2018 describing the effects of smoking on anxiety, and found larger warnings decrease positive pack perceptions and have the potential to reduce the demand for tobacco products (29). Other than this one study, no other countries have adopted this recommendation, and there are no other research testing the effectiveness of such warning labels. Notably, limited empirical research suggests that pictorial mental health warnings for cannabis products are perceived as moderately effective and believable (30). Some evidence suggests that smokers with mental health conditions might respond differently than other populations to tobacco warning messages (31, 32). Smokers with mental health conditions are more likely to perceive physical health warnings as more effective (31), and exhibit greater attention and cognitive responses to health warning labels (32). However, people with mental health conditions are also more likely to avoid looking at the health warning label (32).

Therefore, this study aims to develop novel mental health warning labels for tobacco packaging and to address the following exploratory research questions:

1. Are Mental Health Warning Labels (MHWLs) rated differently to Physical Health Warning Labels (PHWLs) on measures of perceived effectiveness, believability, arousal, valence, acceptability, reactance, novelty of information, and potential effectiveness?
2. Do ratings of warning labels differ according to smoking status or mental health status?
3. Does the difference in ratings between PHWLs and MHWLs vary according to smoking status or mental health status?

## Materials and Methods

The protocol was pre-registered on the Open Science Framework (OSF) (DOI 10.17605/OSF.IO/37X56). Ethical approval was obtained from the Psychology Research Ethics Committee (PREC) at the University of Bath on 27/April/2020 (PREC ID 20-028). Consultation with service users and members of the public has shaped the methodology proposed. Additional information on study methods is provided in Supplementary Material 1.

### Study Design and Setting

This study was an online, randomised experiment with a 2 × 2 × 2 design. Mental health status (people with common mental health disorders vs. people without common mental health disorders) and smoking status (smokers vs. non-smokers) were between subjects’ variables, and type of tobacco health warning (MHWLs vs. PHWLs) was a within subjects’ factor. Warnings were presented in a randomised order, randomised in blocks (with PHWLs and MHWLs constituting each one block) and the order of specific warnings within each block also randomised.

### Participants and Recruitment

Participants were recruited *via* email lists, third-sector services, public engagement events, social media, and PROLIFIC.^1^ Participants were aged 18 years or greater, UK residents, able to read English. We also targeted males when we realised that we had a disproportionate number of females. Our sample is comparable to large scale studies of smokers in the UK in terms of sex, age, and tobacco dependency (33). Smokers were those self-reported to smoke at least 100 cigarettes during their lifetime, and at the time of participating in the survey smoking at least once per week (34). Non-smokers were those self-reported to have smoked at least 100 cigarettes during their lifetime, and at the time of participating in the survey not currently smoking. Having a common mental health condition was defined as scoring above clinical cut-off scores on the GAD-7 (35) and the PHQ-9 (36) [score of ≥ 8 on the GAD-7 (35, 37, 38) and/or ≥ 10 on the PHQ-9 (36)], currently receiving treatment for a mental health problem was not used as grouping criteria.

### Power Calculation

*A priori* power was calculated using G*Power. To achieve 95% power at 5% alpha level to determine a small effect size of f = 0.1 on our primary outcome (effectiveness), we needed 608 participants. A study by Maynard et al. (39) used to guide some measures in this study, examining the difference in perceived effectiveness of tobacco warning labels between smokers and non-smokers, reported a η2 of 0.04, which corresponds to an effect size of f = 0.2 (40). Given that mental health warnings are not established and are untested in this population, we implemented a more conservative effect size for this power calculation (f = 0.1).

### Stimuli

Warning labels were presented as pictorial and text warning together in a stacked format, as in accordance with EU guidance, with a size of 300 by 300 pixels (16).

The final set of MHWLs to be implemented in the online experiment was guided by a patient and public consultancy group. The MHWLs were informed by causal evidence of the effect of smoking on mental health (22–25). The MHWLs were approved by three members of the public with lived experience of smoking and/or mental health in a consultancy focus group, which involved deep discussions around both the text and pictures to be selected for the current study. For more information on development please see the preregistered protocol (DOI 10.17605/OSF.IO/37X56). The MHWLs presented were: “Smoking increases the risk of schizophrenia,” “Smoking harms your mental health,” “Smoking increases the risk of depression,” “Smoking increases anxiety and tension,” “Smoking increases the risk of bipolar disorder” and “Smoking makes stress worse,” due to copyright, stimuli are available on request from the primary author.

PHWLs were selected from set 2 of the European Union pictorial warnings (16). Images from set 2 were chosen due to rotation date occurring at the start of recruitment (May 2020). The following warning labels were selected: “Smoking causes 9 out of 10 lung cancers,” “Smoking increases the risk of blindness,” “Smoking damages your teeth and gums,” “Smoking causes heart attacks,” “Smoking causes stroke and disability,” “Smoking clogs your arteries,” due to copyright, stimuli are available on request from the primary author.

### Primary Outcome Measures

#### Effectiveness

Potential effectiveness of tobacco health warning labels was assessed by a measure adapted from Pechey et al. (41): “Does this affect how much you want to have a cigarette right now?,” answered on a visual 1–7 Likert scale, with 1 labelled as “not at all” and 7 labelled as “very much.” This question was only presented to smokers.

Perceived effectiveness of tobacco health warning labels was assessed by a measure adapted from Maynard et al. (39): “Overall, on a scale of 1–10, how effective is this health warning? (e.g., in encouraging smokers to quit, increasing concerns about smoking, and discouraging youth from starting to smoke)”, with 1 as not at all and 10 as extremely.

### Secondary Outcome Measures

#### Believability

Believability was assessed by asking “Overall, on a scale of 1–10, how believable is this health warning?” The questions was answered on a visual 1–10 Likert scale, with 1 labelled as “not at all” and 10 labelled as “extremely” (39).

#### Valence and Arousal

Emotional response to the health warning labels was assessed using the valence and arousal items of the Self-Assessment Manakin (SAM) (31, 42). Respondents rated their affective states on 9-point visual analogue scales for valence, ranging from 1 “unpleasant” to 9 “pleasant,” and arousal, ranging from 1 “calm” to 9 “agitated,” with 5 as neutral. Note that “agitated” replaced “excited” as this was deemed more appropriate in this study context.

#### Acceptability

Acceptability of tobacco health warning labels was assessed by asking “Do you support or oppose putting this label on tobacco products?” on a visual 1–7 Likert scale, with 1 labelled as “strongly oppose” and 7 labelled as “strongly support.” Adapted from previous research assessing alcohol health warning labels (41). Participants were also asked to provide a response in a free-text box to the question “Why do you support/oppose putting the label on tobacco products?”

#### Reactance

Reactance to health warning labels was assessed using the Brief Measure of Reactance to Health Warnings Scale (RHWS) (43). Respondents were asked “Please state how much you agree or disagree with each statement about the health warning presented above” in response to “The health effect on this warning is overblown,” “This warning is trying to manipulate me” and “This warning annoys me” on a visual 1–5 Likert scale, with 1 labelled as “strongly disagree” and 5 labelled as “strongly agree.” Scores were summed to give an overall total reactance score.

#### Novelty of Information

To assess novelty of information participants were asked: “Have you learned something new from this packaging about the effects of smoking cigarettes on health and wellbeing?” on a visual Likert scale of 1–10 with 1 labelled as “not at all” and 10 labelled as “extremely.” Respondents were then asked to “Please briefly describe your response in the box below.”

#### Qualitative Data

Adapted from Pechey et al. (41), after each block of warning label type participants were presented with an open-text comment box and asked, “Do you have any further thoughts or comments that you would like to add about the last 6 health warnings you viewed?”

#### Additional Measures

We collected data about age, gender, level of education, ethnicity, and country of residence. Smoking status was screened by asking respondents “Have you smoked at least 100 cigarettes in your lifetime?” (Yes/No), and “How often do you smoke cigarettes?” (every day, every week, less than every week or not at all) (34). Fagerström Test of Nicotine Dependence (FTND) was used to assess nicotine dependence of smokers only (44); smokers were asked the type of cigarette smoked (45), and smokers motivation to stop smoking were assessed by the Motivation To Stop Scale (MTSS) (46, 47).

The GAD-7 and PHQ-9 were used to assess having depression or anxiety. For demographic information only participants were also asked if they were receiving treatment for a mental health condition: “Are you currently undergoing treatment (psychological or medical) for a mental health condition?” This question was used to describe the sample characteristics and not for inclusion or grouping criteria.

### Procedure

**FIGURE 1:**
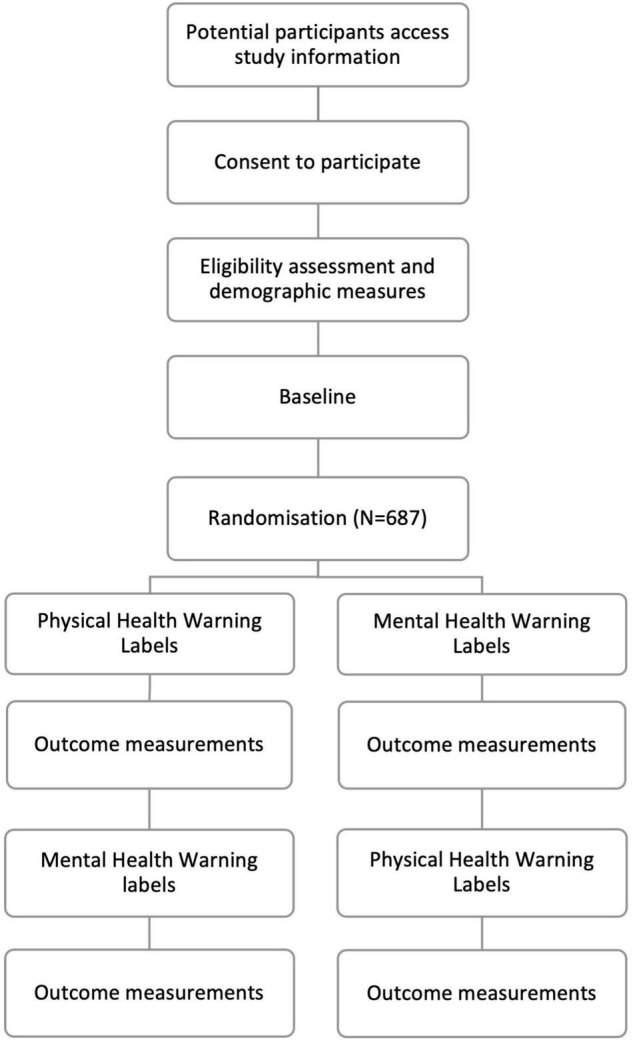
Study flow diagram.

The complete experiment, including screening, consent, and randomisation, was implemented online using Qualtrics.^2^ Following consent, participants completed screening questions and quota items. Participants were asked to rate a series of 12 tobacco health warning labels, 6 of each warning label type (see Figure 1). Participants were debriefed and informed about how be contacted about study findings and/or enter the study prize draw for the chance to win a £50 Amazon Voucher (538 people entered).

### Randomisation

#### Random Allocation and Sequence Generation

Participants were randomly assigned to view either the MHWLs first and then the PHWLs or the PHWLs then the MHWLs with a 1:1 allocation. The order of 6 warnings within each block was also randomised. The random sequence was generated using Qualtrics computer software embedded simple randomisation functions.

#### Allocation Concealment Mechanism

Participants were randomised using Qualtrics, allocation was concealed as the randomised sequence was not recorded and so was unavailable to the research team. The research team were blind to the randomisation order of both blocks and individual warnings within each block.

#### Implementation

Randomisation was implemented using Qualtrics randomisation function, Qualtrics generated the allocation sequence, assigned participants to each order after participants completed consent questions on Qualtrics. Randomisation only occurred after participant identifier, eligibility and consent had been recorded to ensure that implementation was not influenced by the research team or the participants.

### Statistical Analysis

Data were analysed using Stata IC, do-files will be made available on OSF. Dummy variables were generated to indicate participants’ smoking and mental health status. Prior to analyses, composite measures for MHWLs and PHWLs were generated to assess the warning label types, these were created using mean ratings across each health outcome in each label group, for each measure. 2 × 2 × 2 mixed ANOVAs were performed, with mental health condition and smoking status as between-groups factors and health warning label type as a within-groups factor. Label type, mental health status and smoking status were independent variables and perceived effectiveness, believability, arousal, valence, acceptability, reactance, novelty of information were dependent variables.

Free-text questions were manually coded by two authors using content analysis with verbatim responses coded into a small set of meaningful categories. The results of this analysis are reported elsewhere.

#### Missing Data

Forced responses on all primary and secondary measures were implemented *via* Qualtrics to limit missing data. The qualitative, free-text questions were optional responses.

### Protocol Deviations

Midway through the active survey the measure of Potential effectiveness was identified as being without direction and coded incorrectly, therefore was excluded from the analysis. Although we aimed to have balanced groups an error in the Qualtrics survey quota requirements led to unbalanced group sizes.

## Results

### Characteristics of Participants

A total of 687 participants took part in the study, 371 were non-smokers, 316 were smokers, 372 did not have a mental health problem, 315 did have a mental health problem. Across combined groups 219 were non-smokers without mental health problems, 152 were non-smokers with a mental health problem, 153 were smokers without a mental health problem, 163 were smokers with a mental health problem. The mean age was 41.78 (SD = 15.48), and 78.46% (*n* = 539) were females, details of participant characteristics are displayed in Tables 1, 2. Results are presented in Table 3.

**TABLE 1:** Participant characteristics.

Participant characteristic	Mean (SD) or *n* (%) *n* = 687
Age in years	41.78 (15.48)
**Gender**	
Female Male Gender neutral Genderqueer Non-binary Prefer not to say	539 (78.46) 139 (20.23) 1 (0.15) 1 (0.15) 4 (0.58) 3 (0.44)
**Education**	
GCSE or equivalent A-Level or equivalent Undergraduate degree or equivalent Postgraduate degree or equivalent No formal qualifications Prefer not to say	113 (16.45) 162 (23.58) 206 (29.99) 181 (26.35) 20 (2.91) 5 (0.73)
**Ethnicity**	
African Any other Asian Any other mixed/multiple ethnic Any other white Any other ethnic Arab Bangladeshi Caribbean Chinese English/Welsh/Scottish/Northern Irish/British Gypsy or Irish traveller Indian Irish Pakistani Prefer not to say White and Asian White and black caribbean	5 (0.73) 11 (1.60) 6 (0.87) 53 (7.71) 4 (0.58) 1 (0.15) 2 (0.29) 3 (0.44) 24 (3.49) 531 (77.29) 1 (0.15) 15 (2.18) 3 (0.44) 4 (0.58) 7 (1.02) 8 (1.16) 9 (1.31)

**TABLE 2:** Participant mental health and smoking information.

Participant mental health and smoking information	*Mean* (SD) or *n* (%)
Receiving psychological treatment for: Depression Generalised anxiety disorder (GAD) Not receiving treatment Obsessive-compulsive disorder (OCD) Other Panic disorder Phobia Post-traumatic stress disorder (PTSD)	*n* = 681 91 (13.36) 42 (6.17) 481 (70.63) 5 (0.73) 36 (5.29) 7 (1.03) 3 (0.44) 16 (2.35)
**Mental health scores**	
GAD-7 PHQ-9	6.87 (5.45) 8.20 (6.38)
	*n* = 316
Nicotine dependence (mean FTND score)	4.37 (2.73)
**Type of tobacco used**	
Factory made and roll your own Only factory made Only roll your own	81 (25.63) 116 (36.71) 119 (37.66)
**Motivation to stop (MTSS)**	
MTSS score I don’t want to stop smoking I think i should stop smoking but don’t really want to I want to stop smoking but haven’t thought about when I really want to stop smoking but I don’t know when I will I want to stop smoking and hope to soon I really want to stop smoking and intend to in the next 3 months I really want to stop smoking and intend to in the next month	7.04 (1.53) 34 (10.76) 129 (40.82) 35 (11.08) 57 (18.04) 36 (11.39) 18 (5.70) 7 (2.22)

**TABLE 3:** Results of mixed ANOVAs for each outcome.

Outcome		*F*(1, 683)	*p*	η *_*p*_*^2^	95% CI
**Perceived effectiveness**					
	Label type × mental health status × smoking status	0.75	0.39	0.00	0.00, 0.01
	Label type × mental health status	0.11	0.74	0.00	0.00, 0.01
	Label type × smoking status	0.84	0.36	0.00	0.00, 0.01
	Label type	577.64	<0.001**	0.46	0.41, 0.50
	Mental health status	2.09	0.15	0.00	0.00, 0.02
	Smoking status	259.54	<0.001**	0.28	0.22, 0.33
**Believability**					
	Label type × mental health status × smoking status	0.15	0.70	0.00	0.00, 0.01
	Label type × mental health status	1.82	0.18	0.00	0.00, 0.02
	Label type × smoking status	18.37	<0.001**	0.03	0.01, 0.05
	Label type	779.25	<0.001**	0.53	0.49, 0.57
	Mental health status	0.71	0.40	0.00	0.00, 0.01
	Smoking status	144.77	<0.001**	0.17	0.13, 0.22
**Valence**					
	Label type × mental health status × smoking status	0.15	0.70	0.00	0.00, 0.01
	Label type × mental health status	0.50	0.48	0.00	0.00, 0.01
	Label type × smoking status	1.13	0.29	0.00	0.00, 0.13
	Label type	302.59	<0.001**	0.31	0.25, 0.36
	Mental health status	0.15	0.70	0.00	0.00, 0.01
	Smoking status	21.86	<0.001**	0.03	0.01, 0.06
**Arousal**					
	Label type × mental health status × smoking status	0.44	0.51	0.00	0.00, 0.01
	Label type × mental health status	0.17	0.68	0.00	0.00, 0.01
	Label type × smoking status	1.20	0.27	0.00	0.00, 0.01
	Label type	82.34	<0.001**	0.11	0.07, 0.15
	Mental health status	3.12	0.08	0.00	0.00, 0.02
	Smoking status	51.28	<0.001**	0.07	0.04, 0.11
**Acceptability**					
	Label type × mental health status × smoking status	0.00	0.94	0.00	0.00, 1.00
	Label type × mental health status	1.64	0.20	0.00	0.00, 0.02
	Label type × smoking status	0.30	0.58	0.00	0.00, 0.01
	Label type	312.94	<0.001**	0.31	0.26, 0.37
	Mental health status	7.87	0.01*	0.01	0.00, 0.03
	Smoking status	99.31	<0.001**	0.13	0.08, 0.17
**Reactance**					
	Label type × mental health status × smoking status	0.67	0.42	0.00	0.00, 0.01
	Label type × mental health status	1.15	0.28	0.00	0.00, 0.01
	Label type × smoking status	13.53	<0.001**	0.02	0.00, 0.04
	Label type	195.38	<0.001**	0.22	0.17, 0.27
	Mental health status	4.84	0.03*	0.01	0.00, 0.02
	Smoking status	81.22	<0.001**	0.11	0.07, 0.15
**Novelty**					
	Label type × mental health status × smoking status	0.08	0.77	0.00	0.00, 0.01
	Label type × mental health status	0.05	0.82	0.00	0.00, 0.01
	Label type × smoking status	5.86	0.02*	0.01	0.00, 0.03
	Label type	65.27	<0.001**	0.09	0.05, 0.13
	Mental health status	1.16	0.28	0.00	0.00, 0.01
	Smoking status	145.78	<0.001**	0.18	0.13, 0.23

*p < 0.05; **p < 0.001.

### Perceived Effectiveness

There was no significant three-way interaction of label type × mental health status × smoking status for perceived effectiveness. There was no significant interaction of label type with mental health status, or label type with smoking status.

**FIGURE 2:**
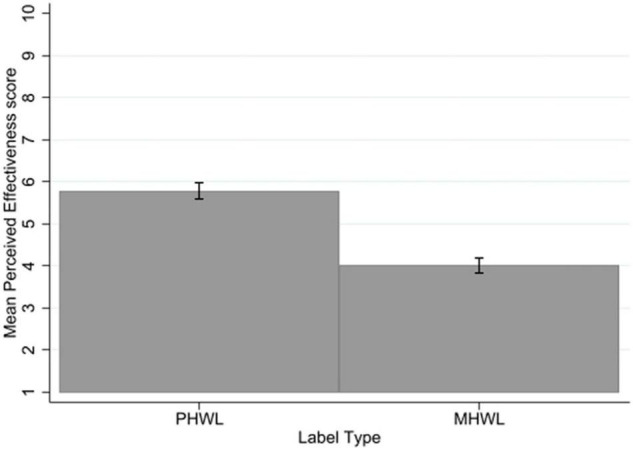
Perceived effectiveness by label type. Error bars represent 95% confidence intervals.

There was a significant main effect of label type on perceived effectiveness with a large effect size (*p* < 0.001, η*_*p*_*^2^ = 0.46) with PHWLs perceived as being more effective (mean = 5.78, SD = 2.55) than MHWLs (mean = 4.02, SD = 2.40) across the sample (Figure 2).

There was no significant main effect of mental health status on perceived effectiveness, people without mental health problems (mean = 5.11, SD = 2.61) did not differ from people with mental health problems (mean = 4.65, SD = 2.61) in their perceptions of effectiveness of the tobacco warning labels. There was a significant effect of smoking status on perceived effectiveness with a large effect size (*p* < 0.001, η*_*p*_*^2^ = 0.28), with non-smokers perceiving labels as more effective (mean = 6.01, SD = 2.32) than smokers (mean = 3.60, SD = 2.34) (see Supplementary Figure 1).

### Believability

There was no significant three-way interaction of label type × mental health status × smoking status for believability. There was no significant interaction of label type with mental health status. There was a significant interaction of label type and smoking status with a small to moderate effect size (*p* < 0.001, η*_*p*_*^2^ = 0.03). Bonferroni corrected *post-hoc t*-tests, indicate that smokers rated MHWLs as less believable than PHWLs (−2.61, SE = 0.17, *p* < 0.001, 95% CI [−3.07, −2.16]) to a greater extent than non-smokers (−1.98, SE = 0.16, *p* < 0.001, 95% CI [−2.31, −1.47]).

**FIGURE 3:**
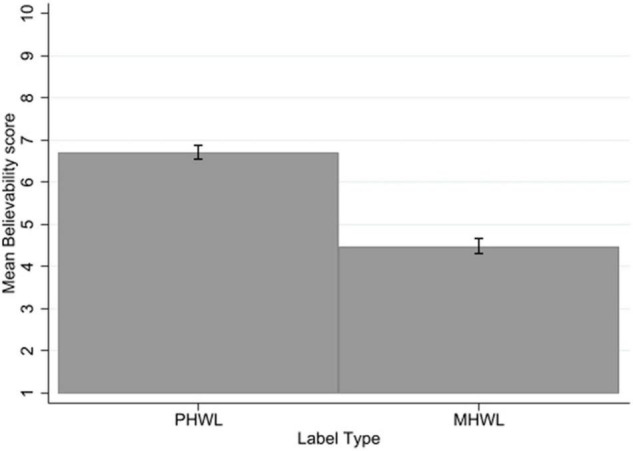
Believability by label type. Error bars represent 95% confidence intervals.

There was a significant main effect of label type on believability with a large effect size (*p* < 0.001, η*_*p*_*^2^ = 0.53) with PHWLs rated as more believable (mean = 6.70, SD = 2.25) than MHWLs (mean = 4.48, SD = 2.44) across the sample (Figure 3).

There was no significant main effect of mental health status on believability, people without mental health problems (mean = 5.73, SD = 2.58) did not differ from people with mental health problems (mean = 5.43, SD = 2.61). There was a significant main effect of smoking status on believability with a large effect size (*p* < 0.001, η*_*p*_*^2^ = 0.17), with non-smokers rating labels as more believable (mean = 6.40, SD = 2.24) than smokers (mean = 4.61, SD = 2.67) (see Supplementary Figure 2).

### Valence

There was no significant three-way interaction of label type × mental health status × smoking status for valence. There was no significant interaction of label type with mental health status, or label type with smoking status.

**FIGURE 4:**
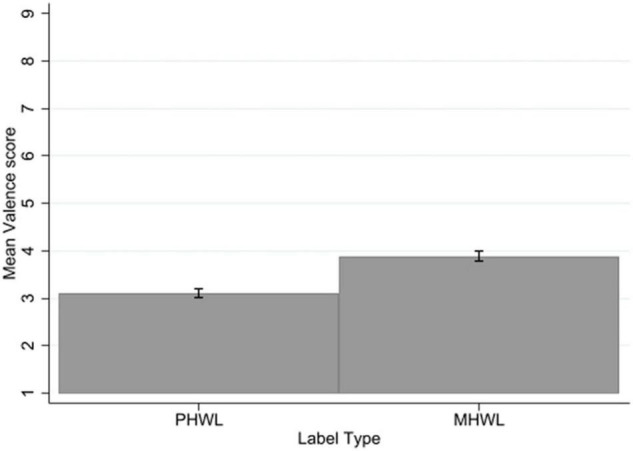
Valence by label type. Error bars represent 95% confidence intervals.

There was a significant main effect of label type on valence, with a large effect size (*p* < 0.001, η*_*p*_*^2.^ = 0.31) with PHWLs rated as more unpleasant (mean = 3.11, SD = 1.26) than MHWLs (mean = 3.89, SD = 1.30) across the sample (Figure 4).

There was no significant main effect of mental health status on valence, people without mental health problems (mean = 3.49, SD = 1.35) did not differ from people with mental health problems (mean = 3.50, SD = 1.33). There was a significant main effect of smoking status on valence, with a small to moderate effect size (*p* < 0.001, η*_*p*_*^2^ = 0.03), with non-smokers rating labels as more unpleasant (mean = 3.31, SD = 1.28), than smokers (mean = 3.72, SD = 1.38) (see Supplementary Figure 3).

### Arousal

There was no significant three-way interaction of label type × mental health status × smoking status for arousal. There was no significant interaction of label type with mental health status or label type with smoking status.

**FIGURE 5:**
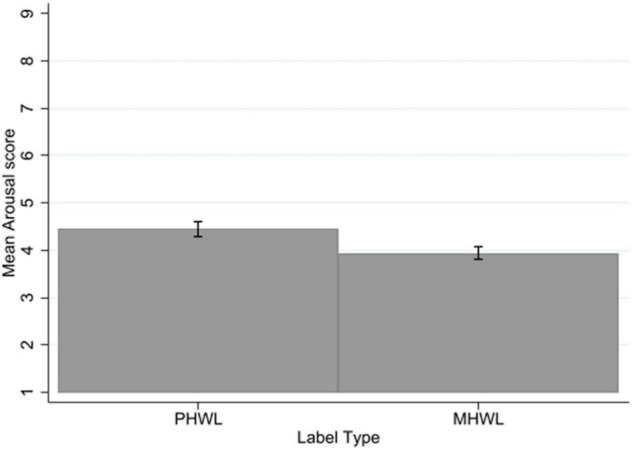
Arousal by label type. Error bars represent 95% confidence intervals.

There was a significant main effect of label type on arousal with a medium to large effect size (*p* < 0.001, η*_*p*_*^2^ = 0.11), with PHWLs were rated as more arousing (mean = 4.45, SD = 1.97) than MHWLs (mean = 3.94, SD = 1.87) across the sample (Figure 5).

There was no significant main effect of mental health status on arousal, people without mental health problems (mean = 4.13, SD = 1.97), did not differ from people with mental health problems (mean = 4.27, SD = 1.90). There was a significant main effect of smoking status on arousal, with a medium effect size (*p* < 0.001, η*_*p*_*^2^ = 0.07), with non-smokers rating labels as more arousing (mean = 4.62, SD = 1.86) than smokers (mean = 3.69, SD = 1.91) (see Supplementary Figure 4).

### Acceptability

There was no significant three-way interaction of label type × mental health status × smoking status for acceptability. There was no significant interaction of label type with mental health status or label type with smoking status.

**FIGURE 6:**
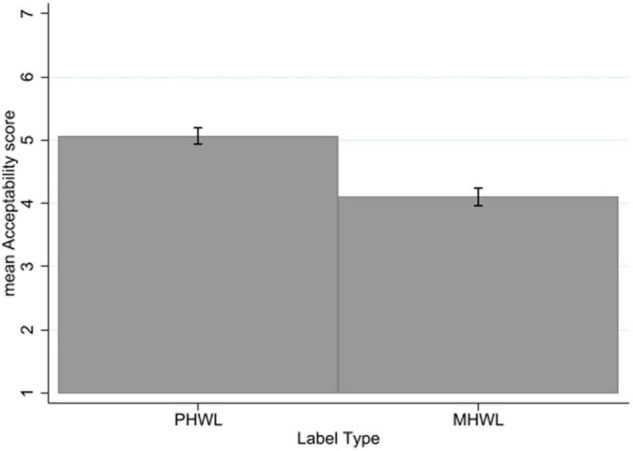
Acceptability by label type. Error bars represent 95% confidence intervals.

There was a significant main effect of label type on acceptability with a large effect size (*p* < 0.001, η*_*p*_*^2^ = 0.31) with PHWLs rated as more acceptable (mean = 5.07, SD = 1.68) than MHWLs (mean = 4.10, SD = 1.80) across the sample (Figure 6).

There was a significant main effect of mental health status on acceptability (*p* = 0.01, η*_*p*_*^2^ = 0.01). People without mental health problems (mean = 4.79, SD = 1.76) rated labels as more acceptable than people with mental health problems (mean = 4.35, SD = 1.83). There was a significant main effect of smoking status on acceptability with a large effect size (*p* < 0.001, η*_*p*_*^2^ = 0.13). Non-smokers rated labels are more acceptable (mean = 5.12, SD = 1.53) than smokers (mean = 3.96, SD = 1.90) (see Supplementary Figure 5).

### Reactance

There was no significant three-way interaction of label type × mental health status × smoking status for reactance. There was no significant interaction of label type with mental health status. There was a significant interaction of label type with smoking status (*p* < 0.001, η*_*p*_*^2^ = 0.02). Bonferroni corrected *post-hoc t*-tests, indicate that smokers rated MHWLs as evoking more reactance than PHWLs (1.62, SE = 0.24, *p* < 0.001, 95% CI [0.98, 2.26]) to a greater extent than non-smokers (0.91, SE = 0.22, *p* < 0.001, 95% CI [0.32, 1.50]).

**FIGURE 7:**
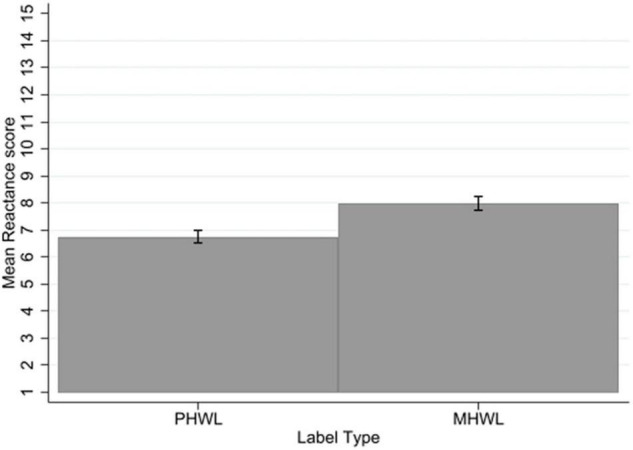
Reactance by label type. Error bars represent 95% confidence intervals.

There was a significant main effect of label type on reactance with a large effect size (*p* < 0.001, η*_*p*_*^2^ = 0.22) with PHWLs evoking less reactance (mean = 6.75, SD = 2.97) than MHWLs (mean = 7.98, SD = 3.41) across the sample (Figure 7).

There was a significant main effect of mental health status on reactance with a small effect size (*p* = 0.03, η*_*p*_*^2^ = 0.01), with greater reactance in people with mental health problems (mean = 7.73, SD = 3.33) compared to those without mental health problems (mean = 7.05, SD = 3.16). There was a significant main effect of smoking status on reactance with a medium to large effect size (*p* < 0.001, η*_*p*_*^2^ = 0.11), non-smokers reported lower reactance (mean = 6.45, SD = 2.86) than smokers (mean = 8.44, SD = 3.36). (see Supplementary Figure 6).

### Novelty

There was no significant three-way interaction of label type × mental health status × smoking status for novelty. There was no significant interaction of label type with mental health status. There was a significant interaction of label type with smoking status (*p* = 0.02, η*_*p*_*^2^ = 0.01). Bonferroni corrected *post-hoc t*-tests, indicate that smokers rated MHWLs similarly in novelty to PHWLs (0.43, SE = 0.17, *p* = 0.08, 95% CI [−0.03, 0.88]), whereas non-smokers rated MHWLs as more novel than PHWLs (0.79, SE = 0.16, *p* < 0.001, 95% CI [0.37, 1.21]).

**FIGURE 8:**
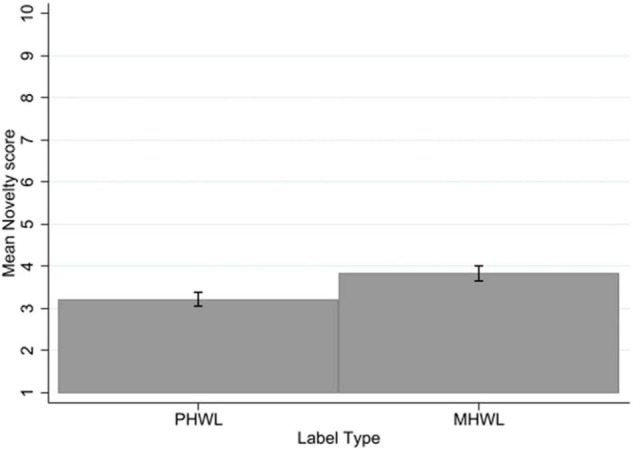
Novelty by label type. Error bars represent 95% confidence intervals.

There was a significant main effect of label type on novelty of information with a medium to large effect size (*p* < 0.001, η*_*p*_*^2^ = 0.09), with PHWLs rated as less novel (mean = 3.21, SD = 2.22) than MHWLs (mean = 3.83, SD = 2.50) (Figure 8).

There was no significant main effect of mental health status on novelty, people without mental health problems (mean = 3.69, SD = 2.49) did not differ in ratings of novelty to people with mental health problems (mean = 3.32, SD = 2.25). There was a significant main effect of smoking status on novelty with a large effect size (*p* < 0.001, η*_*p*_*^2^ = 0.18), with non-smokers rating the labels as more novel (mean = 4.37, SD = 2.34) than smokers (mean = 2.53, SD = 2.03) (see Supplementary Figure 7).

#### Qualitative Responses

The qualitative responses are summarised in depth in another paper. Briefly, respondents displayed mixed support for the mental health warning labels, some respondents supported the mental health warning labels to inform the public about the risks of smoking to mental health and deter smoking, others found the warnings manipulative or thought tobacco warning labels in general were ineffective at preventing smoking. There were also conflicting responses regarding the images used on the warning labels, some described the images depicting mental health as vague, inaccurate, or inappropriate, others described them as accurately representing the mental health condition and being well suited to the text component of the warning. Another key finding was the potential for the mental health warning labels to create stigma for people with mental health conditions. People’s previous understanding or beliefs about smoking were important in their responses, those who believed smoking reduced stress or anxiety seemed to be less supportive of the mental health warning labels.

## Discussion

### Summary of Findings

To our knowledge, this is the first study to design and investigate the effectiveness of a series of mental health tobacco warning labels. We found that MHWLs are perceived as less effective, believable, arousing, unpleasant, and acceptable than PHWLs, but MHWLs evoke more reactance and are rated as more novel. Perceptions of MHWLs did not differ in people with and without mental health problems, except for reactance and acceptability, with greater reactance in people with mental health problems compared to those without mental health problems and people without mental health problems rated labels as more acceptable than people with mental health problems. Perceptions of warning labels differed between non-smokers and smokers. Smokers perceived labels as less effective, believable, arousing, acceptable, novel, more pleasant and had higher reactance. The difference in ratings between PHWLs and MHWLs did not vary according to mental health status. The difference in ratings between PHWLs and MHWLs varied according to smoking status for believability, reactance, and novelty. For believability, differences between MHWLs and PHWLs were greater for smokers than non-smokers, smokers rated MHWLs as much less believable than PHWLs. For reactance, differences between MHWLs and PHWLs were greater for smokers than non-smokers, smokers rated MHWLs as evoking much more reactance than PHWLs. For novelty, smokers rated MHWLs similarly in novelty to PHWLs, whereas non-smokers rated MHWLs are more novel than PHWLs.

### Strengths and Limitations

Strengths of our study include the large sample size, inclusion of both smokers and non-smokers, and people with and without mental health problems. Another strength is the use of patient and public involvement (PPI) throughout the study design, including development of the warnings and survey measures. Limitations include the use of self-report measures, Tamayo et al. (48) suggest that explicit reactions could be different to implicit reactions to warning labels, thus self-report measures may not accurately reflect people’s true perception of the warning labels (48). The use of self-report measures also means it is unclear whether MHWLs influence actual effectiveness or smoking behaviour, although a meta-analysis found that perceived effectiveness does predict quit intentions and cessation (49). The study also includes only those with common mental health conditions, depression and anxiety, in the mental health group, and not people with more severe and complex psychotic spectrum disorders, this limits the generalisability of the findings to a wider population of people with more severe mental health problems. Also, not including this population in our sample could have affected our findings as people with more severe psychotic spectrum disorders could have different perceptions of the MHWLs compared to people with more common mental health conditions (31). The study is also limited by its sample which is not representative of the wider population. Our sample was made up of mostly white females; research suggests that both gender and ethnicity can influence ratings of health warning labels, with females rating labels as more effective, and people of white ethnicity rating labels as less effective (50, 51). Future research should aim to increase the representativeness of the sample and investigate potential moderating effects of gender, and ethnicity on responses to mental health warning labels.

### Interpretation and Comparison to Other Studies

MHWLs could be perceived as less effective due to the causal language used. PHWLs used the phrase “smoking causes” whereas MHWLs used “smoking increases the risk of.” Our PPI focus group advised us to use the phrase “increases risk of” as “causes” was viewed as reductionist and potentially stigmatising. However, evidence suggests that warnings with strong causal language are perceived as most effective at discouraging people to smoke, thus the lack of causal language in the MHWLs could have limited their effectiveness (17, 52). Future research needs to investigate how to balance the need for MHWLs to be effective and the potential for mental health stigma.

How graphic the images were could explain the differences between the warning labels, as PHWLs had more graphic images than the MHWLs. Research suggests that graphic images increase perceived harms of smoking, quit intentions, prevention of smoking and are more effective (17, 53–59). By their nature, the mental health images were less graphic than those included in the PHWL condition (e.g., surgical scars, tooth decay). It is possible that this influenced the rating of MHWLs as more pleasant and less arousing. This is supported by some qualitative feedback such as “*Think there are better images to convey poor mental health. Feel quite calm about this image, even though have struggles with my mental health this image and message doesn’t really affect me.*” However, capturing mental health problems as a single image, particularly a graphic image, is very challenging. Not only is it difficult to represent mental health problems in a picture but doing so raises ethical issues. Negative media images of mental health problems can elicit mental health stigma and can impair the self-esteem and recovery of people with mental health problems (60). Thus, ethically representing mental health images graphically is a challenge. Arguably, text-only warnings could be used to address this challenge, however text-only warnings are not demonstrated to be as effective as pictorial warnings in the existing literature (30). Pictorial warnings have also been found to be important for communicating the effects of smoking in low and middle income countries with low literacy rates (45, 61). Future research should include people with mental health problems to further develop the images on mental health warning labels.

The “misattribution hypothesis” could also explain some of the differences seen between MHWLs and PHWLs. There is a common misperception that smoking can alleviate stress and help people to cope in challenging situations (19, 27). Many people also describe using smoking as a method to “self-medicate” mental health symptoms, such as depression or anxiety (19, 27, 62). This view is persistent among many populations, including health professionals (20, 21). Therefore, the MHWLs contradict peoples’ current understanding of the effects of smoking, and the effects of smoking on mental health are not well understood. This contrasts to PHWLs, which are well understood and communicated from government tobacco control policies, including tobacco warning labels (63). Considering the misattribution hypothesis, the MHWLs are at odds with smokers own experience of smoking, compared to non-smokers who do not experience the effects of smoking, which could explain why smokers rated the MHWLs as less believable and evoking more reactance than PHWLs, to a greater extent than non-smokers (19, 27, 62).

There could also be more defensive reactions to the MHWLs compared to PHWLs as MHWLs challenge and threat people’s current beliefs about smoking and mental health (19, 27, 62). This is supported by initial feedback from our qualitative data, such as: *“I think for a lot of people smoking actually helps with anxiety and tension. This seems like a lie.”; “I don’t believe this is true.”* Another issue which could explain this is the threat of stigma. Qualitative data collected from this study suggests that participants found the MHWLs to be reductive and placing blame upon the individual for their mental health problem: *“This sounds odd and feels a bit unpleasant (mental health is serious and doesn’t need more stigma! if I was depressed the last thing I would want to hear it’s that I am depressed because I smoke)”*; *“It seems more likely to increase the risk of depression. But again, there’s the risk of people blaming depressed people for being depressed just because they smoke”; “Mental health already is stigmatised against, without these blaming statements.”* Thus the MHWLs cause a threat, which could explain the higher reactance and less acceptance, particularly in people with mental health problems (64–66). Future research should further investigate how to balance the potential stigma of MHWLs against using them as an effective tool for health risk communication.

Consistent with the physical health warning literature we found that ratings differed according to smoking status. In line with the literature, smokers perceived labels as less effective, believable (14, 39, 67), arousing (48, 68), acceptable and novel (69). Smokers rated HWLs as more pleasant (higher valence) (48), and had higher reactance to the HWLs (70, 71). These differences could be explained by perceived susceptibility to the warning labels, as previous research has found the higher the perceived susceptibility to the HWL, the higher the ratings of effectiveness and believability (17, 32). Smokers are known to judge the risk of health effects of smoking as lower than non-smokers, potentially because they minimise the risk to themselves and so rate the labels as less effective and believable (72). Perceived susceptibility is also important in determining fear responses to HWLs, this could explain why smokers exhibited less arousal, and rated warnings as more pleasant (65, 73). Higher exposure to tobacco health warnings and information on the health effects of smoking could explain why smokers rated all warning labels as less novel (63, 74). Smokers’ rating of HWLs as less acceptable and having higher reactance could be explained by cognitive dissonance experienced when viewing the labels. Smokers are aware of the health risks of smoking but continue to smoke, which is aversive, so to minimise this smokers avoid, ignore or reject HWLs, evoking higher reactance and lower ratings of acceptability (34, 70, 71, 75, 76).

We found that ratings of warning labels did not differ according to mental health status except for reactance and acceptability, with greater reactance in people with mental health problems compared to those without mental health problems and people without mental health problems rated labels as more acceptable than people with mental health problems. Research on differences in responses to tobacco warning labels in people with and without mental health problems is limited and conflicting. Our findings contrast with some previous findings, Coletti et al. (31) assessed views of young people with recent onset psychosis (ROP) of physical health warning labels and found that people with ROP were more likely to rate the warning labels as effective than healthy controls. However, our findings are similar to Osman et al. (32) who found that although at first introduction of PHWLs people with and without mental health problems differed in their responses, over time responses increased in people with low depression symptoms and the difference between mental health groups disappeared. Osman et al. (32) assessed depression using the Epidemiological Studies Depression scale (CES-D-7), which is similar to our assessment of depression. One explanation for the difference in findings between studies is that Coletti et al. (31) used clinical assessments of psychotic disorders whereas our study looked at symptoms of depression and anxiety reaching the threshold for caseness. It could be that severity of mental health symptoms, or differences in mental health disorders and measurement tools influenced responses to warning labels. Another explanation could be age, Coletti et al. assessed responses in young people (mid-20s) whereas this study and Osman assessed responses in adults (40s). It could be that age is important in predicting differences in responses in people with and without mental health status, which is a topic for future research.

### Implications for Policy and Practice

To our knowledge, this is the first study to design and test the perceptions of a series of MHWLs for tobacco. MHWLs were identified as low to moderately effective method for the communication of health risks of smoking on mental health, however, refinement of the MHWLs is necessary. Future research should further refine the MHWLs to provide novel information to inform the public about an underappreciated health risk of smoking, whilst balancing the risk of stigmatising mental health problems. Future research could also investigate whether communicating the benefits of smoking cessation for mental health *via* tobacco warning labels is effective, such gain-framed appeals are suggested to be effective for smoking abstinence (26, 77). It appears that the same underlying mechanisms are present for MHWLs as PHWLs, in terms of differences in perceptions for smokers and non-smokers, future research should investigate whether susceptibility to the mental health risks of smoking influences responses. However, much of the health warning label literature is conducted in developed and high-income countries, although more work is being done in developing countries the evidence is more limited and implementation of warnings more challenging (78), this has implications for the design and potential implementation of MHWLs, thus future research should investigate MHWLs in developing countries. When designing this study we found large variation in the outcomes measured and measurement tools in warning label research, and so we recommend that a Core Outcome Set be developed for warning label research (79).

## Conclusion

Mental health warnings labels could be an effective means to communicate the effects of smoking on mental health. MHWLs are perceived as less effective, believable, arousing, unpleasant, and acceptable than PHWLs, but MHWLs evoke more reactance and are rated as more novel. Perceptions of MHWLs did not differ in people with and without mental health problems except for reactance and acceptability, but consistent with the PHWL literature, perceptions of MHWLs differed between non-smokers and smokers.

## Data Availability Statement

The datasets presented in this article are not readily available because anonymised data can be accessed from University of Bath’s Research Data Archive. All data will be anonymised using unique identifiers, and data access will be restricted. Data will be made available to approved bona-fide researchers: after they have signed a data access agreement, the person will be granted access to the University of Bath’s Data Archive. Requests to access the datasets should be directed to https://library.bath.ac.uk/research-data/archiving-and-sharing/home.

## Ethics Statement

The studies involving human participants were reviewed and approved by the Psychology Research Ethics Committee (PREC) at the University of Bath. The patients/participants provided their written informed consent to participate in this study.

## Author Contributions

KS and GT involved in study conception. KS, CB, and RN involved in acquisition, analysis, and interpretation of the data. KS led the project and drafted the manuscript. All authors were involved in the design and revising the manuscript.

## Conflict of Interest

GT had previously received funding from Grand (Pfizer) for work not related to this project. The remaining authors declare that the research was conducted in the absence of any commercial or financial relationships that could be construed as a potential conflict of interest.

## Publisher’s Note

All claims expressed in this article are solely those of the authors and do not necessarily represent those of their affiliated organizations, or those of the publisher, the editors and the reviewers. Any product that may be evaluated in this article, or claim that may be made by its manufacturer, is not guaranteed or endorsed by the publisher.

## Footnotes

1
https://prolific.co/

2
www.qualtrics.com
