# Supplementary material for: Effectiveness of Mental Health Warnings on Tobacco Packaging in People With and Without Common Mental Health Conditions: An Online Randomised Experiment
Source: Front Psychiatry. 2022 Jul 14;13:869158. doi: 10.3389/fpsyt.2022.869158 (PMC9331922; doi:10.3389/fpsyt.2022.869158)

Table of Contents

[Supplementary materials 1 1](#_Toc94817093)

[Supplementary Materials 2 3](#_Toc94817094)

[Figure 1 Perceived Effectiveness by smoking status and mental health status. 3](#_Toc94817095)

[Figure 2 Believability by smoking status and mental health status. 3](#_Toc94817096)

[Figure 3 Valence by smoking status and mental health status 3](#_Toc94817097)

[Figure 4 Arousal by smoking status and mental health status 4](#_Toc94817098)

[Figure 5 Acceptability by smoking status and mental health status 4](#_Toc94817099)

[Figure 6 Reactance by smoking status and mental health status 5](#_Toc94817100)

[Figure 7 Novelty by smoking status and mental health status 6](#_Toc94817101)

# **Supplementary materials 1**

Methods

Additional measures

The GAD-7 and PHQ-9 were used to assess having depression or anxiety. The GAD-7 is a seven-item, self-report measure assessing experience of anxiety symptoms in the previous 2 weeks on a scale of 0 ‘not at all’ to 3 ‘nearly every day’ (1). The PHQ-9 is a nine-item, self-report measure assessing experience of depression symptoms in the previous 2 weeks on the same 0 - 3 scale (2). Participants were also asked if they are receiving treatment for a mental health condition “Are you currently undergoing treatment (psychological or medical) for a mental health condition. one of the following conditions: depression, generalised anxiety disorder (GAD), panic disorder, obsessive-compulsive disorder (OCD), post-traumatic stress disorder (PTSD), simple phobias, other (please specify)”. Adapted from NICE (2011) list of common mental health disorders (3).

Procedure

Warnings remained visible on the screen throughout the completion of outcome measures. After rating responses for each health warning label block, participants were given the option for further comments in a free-text box. Participants were debriefed and informed about how be contacted about study findings and/or enter the study prize draw for the chance to win a £50 Amazon Voucher (538 people entered).

Statistical Analyses

Prior to analyses, composite measures for MHWLs and PHWLs were generated to assess the warning label types, these were created using mean ratings of each warning label (depression, anxiety etc) for each measure. 2 x 2 x 2 mixed ANOVAs were performed, with a significance level of *p* < .05. Effect sizes are reported as Partial *η^2^*, with *η_p_^2^* = .01 was considered small, *η_p_^2^* = .06 moderate, and *η_p_^2^*  = .14 a large effect (4). The data were screened for outliers using box plots, but no extreme outliers were identified.

Data were checked for normality of standardised residuals using histograms and Shapiro-Wilks test, although minor deviations were identified there were no substantial deviations from normality. Homogeneity of variance was tested with Levene’s test, homogeneity of variances was violated for believability, acceptability, reactance and novelty. Homogeneity of variance-covariance matrices was assessed using Box's M, homogeneity of variance-covariance was violated for believability, acceptability, reactance, and novelty. Transformation of the data could be recommended, however given that ANOVAs are demonstrated to be robust against violations of both normality and homogeneity, and that transformation could reduce power and make interpretation difficult, the data are not transformed, instead results should be interpreted with caution (4–6).

**REFERENCES**

1. Spitzer RL, Kroenke K, Williams JBW, Löwe B. A Brief Measure for Assessing Generalized Anxiety Disorder: The GAD-7. Arch Intern Med. 2006 May 22;166(10):1092.

2. Kroenke K, Spitzer RL, Williams JBW. The PHQ-9: Validity of a brief depression severity measure. J Gen Intern Med. 2001 Sep;16(9):606–13.

3. National Collaborating Centre for Mental Health (UK). Common Mental Health Disorders: Identification and Pathways to Care. Leicester (UK): British Psychological Society; 2011.

4. Tabachnik BG, Fidell LS. Using Multivariate Statistics. 6th ed. Northridge; 2013.

5. Blanca MJ, Alarcón R, Arnau J. Non-normal data: Is ANOVA still a valid option? Psicothema. 2017 Nov;(29.4):552–7.

6. Schmider E, Ziegler M, Danay E, Beyer L, Bühner M. Is It Really Robust?: Reinvestigating the Robustness of ANOVA Against Violations of the Normal Distribution Assumption. Methodology. 2010 Jan;6(4):147–51.

# **Supplementary Materials 2**

## Figure 1 Perceived Effectiveness by smoking status and mental health status.


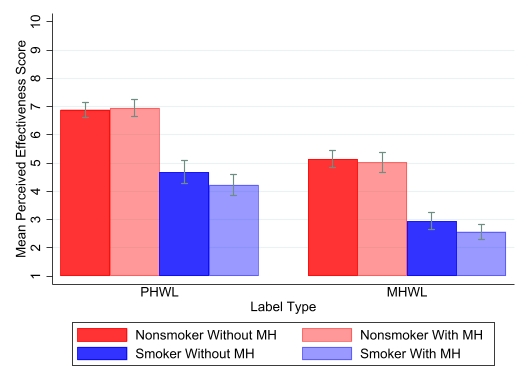


## Figure 2 Believability by smoking status and mental health status.


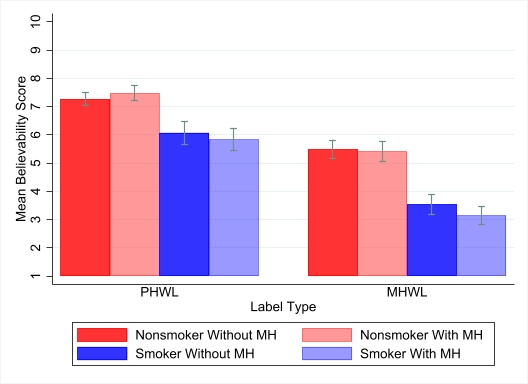


## Figure 3 Valence by smoking status and mental health status


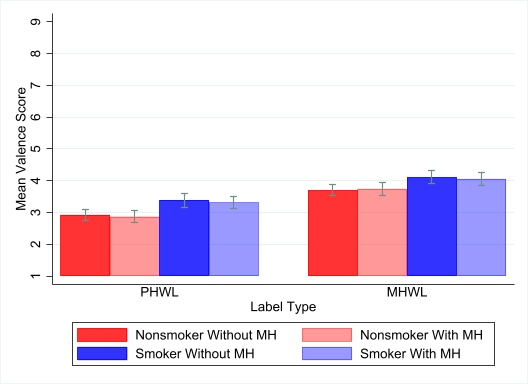


## Figure 4 Arousal by smoking status and mental health status


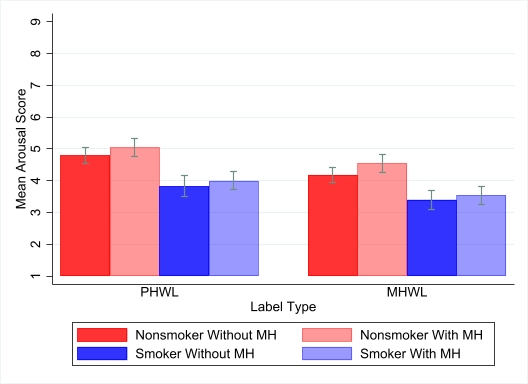


## Figure 5 Acceptability by smoking status and mental health status


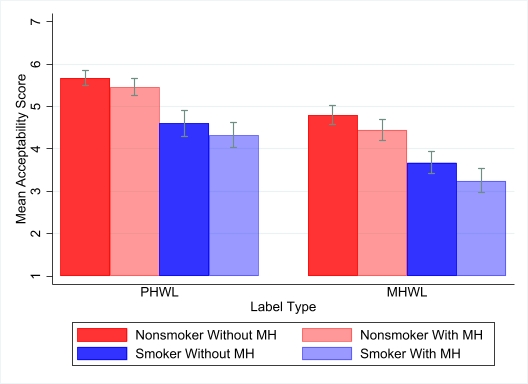


## Figure 6 Reactance by smoking status and mental health status


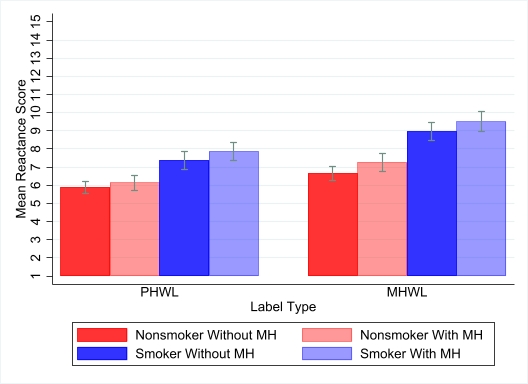


## Figure 7 Novelty by smoking status and mental health status


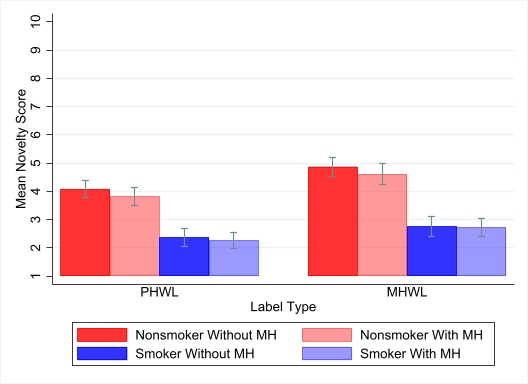

Supplement: Supplementary file 1 [file Table_1.DOCX]
